# Outcome of Primary Nonpenetrating Deep Sclerectomy in Patients with Steroid-Induced Glaucoma

**DOI:** 10.1155/2018/9215650

**Published:** 2018-04-04

**Authors:** Abdelhamid Elhofi, Hany Ahmed Helaly

**Affiliations:** Ophthalmology Department, Faculty of Medicine, Alexandria University, Alexandria, Egypt

## Abstract

**Purpose:**

To evaluate the outcome of primary nonpenetrating deep sclerectomy (NPDS) in patients with steroid-induced glaucoma.

**Methods:**

This was a retrospective interventional clinical study that included 60 eyes of 60 steroid-induced glaucoma patients that had undergone NPDS. Patients were followed up for 4 years. Data from the records was retrieved as regards corrected distance visual acuity (CDVA), intraocular pressure (IOP), visual field mean defect (dB), and number of antiglaucoma medications needed if any. Complete success of the surgical outcome was considered an IOP ≤ 21 mmHg with no antiglaucoma medications. Qualified success was considered an IOP ≤ 21 mmHg using antiglaucoma medications.

**Results:**

The mean age was 21.2 ± 8.5 years (ranged from 12 to 35 years). At 48 months, mean IOP was 13.6 ± 2.8 mmHg (range 11–23 mmHg). This represented 60% reduction of mean IOP from preoperative levels. One case had YAG laser goniopuncture. Three cases required needling followed by ab interno revision. Using ANOVA test, there was a statistically significant difference between preoperative and postoperative mean IOP values (*P* = 0.032). Twelve, 16, and 20 patients required topical antiglaucoma medications at 24, 26, and 48 months postoperative, respectively.

**Conclusion:**

Primary nonpenetrating deep sclerectomy is a safe and an effective method of treating eyes with steroid-induced glaucoma. No major complications were encountered. After 4 years of follow-up, complete success rate was 56.7% and qualified success rate was 70%.

## 1. Introduction

Steroid-induced glaucoma (SIG) is considered as a secondary open-angle glaucoma. It can occur with topical, periocular, or systemic routes of administration of steroids [1, 2]. Like primary open-angle glaucoma, the main problem is pathology of the trabecular meshwork. The exact pathogenesis is not fully understood, but it is suggested that corticosteroids decrease the conventional trabecular outflow and thus increase the intraocular pressure (IOP). Corticosteroids cause accumulation of glycosaminoglycans in the trabecular meshwork, which leads to increasing outflow resistance [3–6].

The MYOC gene is equally expressed in the trabecular meshwork cells. Normal myocilin increases in response to trabecular meshwork stress, increased IOP, or dexamethasone, suggesting it has a protective role. Mutations in this gene cause the formation of an abnormal TIGR/MYOC protein that causes trabecular meshwork (TM) clogging and increases outflow resistance [6–8]. Also, the effect of steroids on inhibiting phagocytosis of TM endothelial cells and decreasing the expression of extracellular proteinases may play a role in the pathogenesis of SIG [9–11].

Nonpenetrating deep sclerectomy (NPDS) is a nonpenetrating glaucoma procedure that involves the removal of a deep scleral flap [12, 13]. This leads to the formation of a scleral space where the aqueous humor is collected and then drained either to the subconjunctival space or to the suprachoroidal space. It works by deroofing Schlemm's canal (SC) and the removal of juxtacanalicular trabecular meshwork as well as a part of the corneoscleral trabecular meshwork [14–17]. The main resistance to aqueous humor outflow lies in those membranes. Yet, NPDS maintains an intact part of the TM which allows gradual decrease in the IOP preventing the sudden hypotony that occurs with the penetrating glaucoma surgery. Space-maintaining devices, such as collagen and hydrophilic acrylic implants, are used with the NPDS to maintain the scleral space during the period of maximal healing. This augments the IOP lowering effect of the surgery [18, 19].

The aim of the current study was to evaluate the outcome of primary nonpenetrating deep sclerectomy in patients with steroid-induced glaucoma.

## 2. Subjects and Methods

This was a retrospective interventional clinical study that included 60 eyes of 60 steroid-induced glaucoma patients. Records of the patients were revised, and the patients were recalled for a final follow-up visit. The included patients were nonresponsive or had intolerance to maximal medical treatment and had undergone a primary nonpenetrating deep sclerectomy with a collagen implant. All cases were operated upon by the same surgeon (A.E.). Included patients had complete records covering a follow-up period of at least 4 years. Patients were excluded if they had other ocular pathology or comorbidity, for example, had complicated cataract, had congenital glaucoma, or had previous laser or surgical intervention before NPDS. Patients with incomplete data or follow-up period were also excluded.

The current study was approved by the local ethics committee of the Faculty of Medicine, Alexandria University, Egypt. Tenets of the Declaration of Helsinki were followed. All patients signed an informed consent at the final follow-up visits.

### 2.1. Surgical Technique

The cases were operated upon using general anesthesia. A fornix-based superior conjunctival flap was done. Dissection of a superficial scleral flap (one-third of the scleral depth) was continued until a clear cornea is reached anteriorly; the dimensions of the rectangular flap were 5 × 5 mm. This was followed by the application of 0.02% of mitomycin C for 2 minutes using soaked sponges under the scleral flap and the conjunctiva, then a thorough wash with a balanced salt solution. A 4 × 4 mm deeper scleral flap was dissected anteriorly to Schlemm's canal and continued further anterior in the cornea to create a trabeculo-Descemet's window. The deep scleral flap was excised. A collagen implant was used to maintain the space created and fixed with a single 10-0 suture. The superficial scleral flap was then sutured using 10-0 nylon sutures. The conjunctiva was sutured with 8-0 absorbable sutures. Postoperative topical antibiotic and steroid were prescribed 5 times daily for the first month, and then steroid eye drops were tapered slowly. Any complications were recorded. Also, any further intervention needed was recorded such as yttrium aluminum garnet (YAG) laser goniopuncture, needling, or ab interno revision plus subconjunctival 5-fluouracil injection.

Needling was performed under topical anesthesia using 27-gauge needle. The needle was used to dissect adhesion under the superficial scleral flap. At the end of the procedure, subconjunctival injection of 0.5 ml of 5-flourouracil was done. The technique of ab interno revision was as follows: In the operating room under topical anesthesia using methyl cellulose injection in the anterior chamber through a side port, a spatula was inserted either temporally or nasally until the tip of the spatula is seen under the conjunctiva doing synechiolysis with a sweeping to and fro movement attacking the contralateral side of the scleral flap. This was followed by removing the methyl cellulose by washing the anterior chamber by balanced saline solution. This would form the bleb ensuring success of the procedure converting it into a penetrating trabeculectomy. Finally, subconjunctival injection of 0.5 ml of 5-flourouracil was done.

Patients were followed up for 4 years. Data from the records was retrieved as regards corrected distance visual acuity (CDVA), IOP, visual field mean defect (dB), and number of antiglaucoma medications needed if any.

Corrected distance visual acuity was measured and expressed in logMAR units for statistical analysis preoperative and up to 48 months following surgery. Rules mentioned by Holladay [20] for calculating average visual acuity were followed. Haag-Streit Goldmann applanation tonometer AT 900 (Haag-Streit, Berne, Switzerland) was used for IOP measurements. Intraocular pressure measurements were taken while the patient was seated at a slit lamp. All measurements were obtained during the daily office work hours by the same operator to minimize the variations. An average of 3 reliable measurements was recorded. Care was taken to avoid squeezing of the eyelids to avoid false rise in the IOP. Visual field testing was done using Humphrey field analyzer (Humphrey Instruments, San Leandro, Calif) automated perimetry. The 24-2 test was performed using white stimulus on white background. Patients were instructed before taking the visual field exam. The first visual field test result was discarded to avoid the learning curve effect. All visual field tests were done by the same operator. Also, an average visual field mean defect of 3 reliable tests was recorded. Unreliable visual field tests (e.g., presence of artifacts or sleepy patients) were excluded and repeated.

Complete success of the surgical outcome was considered an IOP ≤ 21 mmHg with no antiglaucoma medications. Qualified success was considered an IOP ≤ 21 mmHg using antiglaucoma medications. Failure was defined as an IOP > 21 mmHg despite the use of medications, an IOP < 4 mmHg (hypotony), or a need for another glaucoma intervention was present.

Data analysis was performed using the software SPSS for Windows version 20.0 (SPSS Inc., Chicago, USA). Quantitative data were described using range, mean, and standard deviation. Normality of data samples was evaluated using the Kolmogorov-Smirnov test. ANOVA test was used to compare between different means. Paired *t*-test was used for comparisons between means of the preoperative and postoperative data. Kaplan-Meier method was used for survival analysis to report complete success and qualified success percentages along time. Chi-square test was used to compare between different percentages. Differences were considered statistically significant when the associated *P* value was less than 0.05.

## 3. Results

The study included 60 eyes of 60 patients. Thirty-four patients (57%) were males and 36 patients (43%) were females. The mean age was 21.2 ± 8.5 years (ranged from 12 to 35 years). Most of the patients were exposed to steroids for a long duration of time (6 months or more). Prolonged use of topical steroids was the cause in 52 patients representing 87% of the cases while prolonged use of systemic steroids was the cause in 8 patients representing 13% of the cases.

The mean preoperative CDVA was 0.37 ± 0.13 logMAR (range 0.1–0.7 logMAR). The mean preoperative IOP was 34.22 ± 6.90 mmHg (range 27–45 mmHg). The mean deviation of preoperative visual field defect was −12.51 ± 7.33 dB (range −23.44 to −2.2 dB). The average number of preoperative anti glaucoma medications was 2.47 ± 0.51.

Mean postoperative IOP at 6 months was 12.3 ± 2.1 mmHg (range 8–15 mmHg). This represented 64% reduction of mean IOP from preoperative levels. No cases needed YAG laser goniopuncture or ab interno revision. Two cases had needling with subconjunctival injection of 5-fluorouracil. At 12 months, mean IOP was 12.3 ± 2.6 mmHg (range 8–17 mmHg). Two patients needed YAG laser goniopuncture. Three cases required needling and no cases needed ab interno revision. Mean postoperative IOP at 24 months was 12.8 ± 3.2 mmHg (range 9–18 mmHg). Another two patients needed YAG laser goniopuncture. Three cases required needling and one case needed ab interno revision. Mean postoperative IOP at 36 months was 13.3 ± 3.1 mmHg (range 10–23 mmHg). No other cases had YAG laser goniopuncture. Two cases required needling followed by ab interno revision. At 48 months, mean IOP was 13.6 ± 2.8 mmHg (range 11–23 mmHg). This represented 60% reduction of mean IOP from preoperative levels. One case had YAG laser goniopuncture. Three cases required needling followed by ab interno revision. Using ANOVA test, there was a statistically significant difference between preoperative and postoperative mean IOP values (*P* = 0.032). Using paired *t*-test to compare each postoperative mean IOP to the preoperative levels, the difference was statistically significant (*P* < 0.001) (Table 1). Using paired *t*-test to compare final 48 months mean IOP with previous means, it was significantly higher than that of 6 months, 12 months, and 24 months (*P* < 0.05) but was not statistically different from that of 36 months (*P* = 0.621).

**Figure 1:**
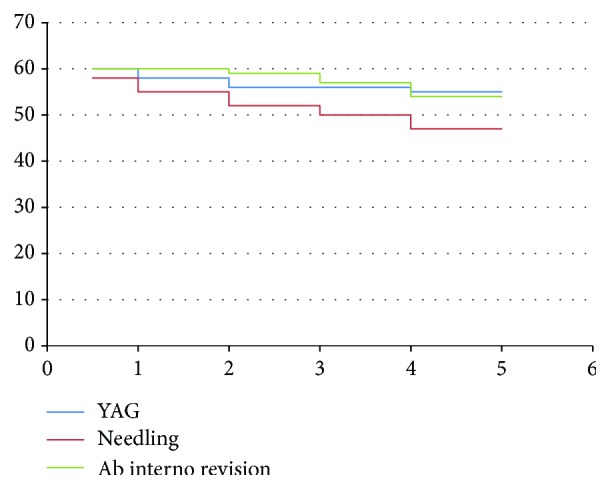
Survival analysis for YAG laser goniopuncture, needling, and ab interno revision. *y*-axis: number of patients; *x*-axis: time interval in years.

Total number of cases that required YAG laser goniopuncture along the 48 months follow-up was 5 cases. As regards needling with subconjunctival injection of 5-fluoruouracil, it was done in 13 cases. Ab interno revision was required in 6 cases.  Figure 1 shows a survival analysis curve for YAG laser goniopuncture, needling, and ab interno revision. No major postoperative complications were detected. However, cheese wiring of the conjunctival sutures was detected in 8 cases.

Four patients required topical antiglaucoma medications by the end of the 6th month. By the end of the 1st year, eight patients in total required antiglaucoma medications. Twelve, 16, and 20 patients required topical antiglaucoma medications at 24, 26, and 48 months, respectively.  Table 2 shows the mean values for topical antiglaucoma medications required at different time intervals. Using ANOVA test, there was a statistically significant difference between preoperative and postoperative mean number of topical medications required (*P* = 0.021). Using multiple paired *t*-tests, mean number of topical medications required was significantly higher than the mean number of medications required at 6 months, 12 months, 24 months, and 36 months.

Table 3 shows the mean preoperative and postoperative CDVA values. Using ANOVA test, there is no statistically significant difference between different means (*P* = 0.105). Using paired *t*-test, there is no statistically significant difference between preoperative mean CDVA and postoperative levels at 6 months, 12 months, 24 months, 36 months, and 48 months. Table 3 shows the mean preoperative and postoperative visual field defect mean deviation. There is a statistically significant difference between the different means using ANOVA test (*P* = 0.021). Visual field defect mean deviation at 24 months, 36 months, and 48 months was statistically significantly higher than that of preoperative level.

**Figure 2:**
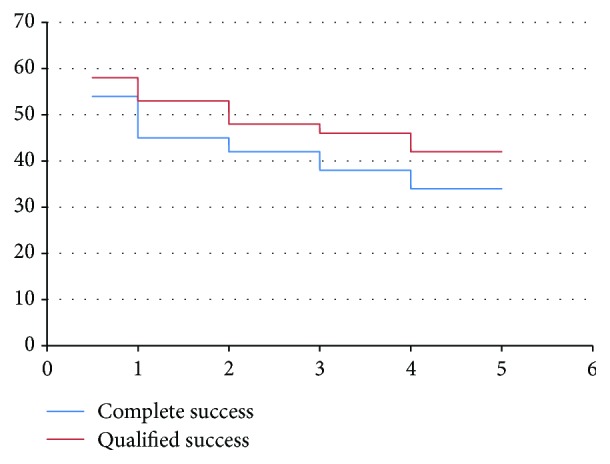
Survival analysis curve for complete and qualified success rates. *y*-axis: number of patients; *x*-axis: time interval in years.

Complete success rate at 6 months was 90% (54 eyes), and qualified success rate at 6 months was 96.7% (58 eyes). Complete success rate was 75% (45 eyes), 70% (40 eyes), and 63.3% (38 eyes) at 12 months, 24 months, and 36 months, respectively. Qualified success rate was 88.3% (53 eyes), 80% (48 eyes), and 76.7% (46 eyes) at 12 months, 24 months, and 36 months, respectively. At 48 months, complete success rate was 56.7% (34 eyes), and qualified success rate was 70% (42 eyes).  Figure 2 shows survival curves for complete and qualified success rates at different time intervals.

## 4. Discussion

Steroid-induced glaucoma can theoretically occur after any route of administration of steroids. This can be troublesome in some cases when the discontinuation of steroid is harmful or when we cannot remove the already injected steroids, for example, in the case of intravitreal injection or the use of an intravitreal dexamethasone implant. The two main routes of administration in the current study were either topical route especially in cases of allergy and post laser vision correction surgery or systemic route in cases of immune disorders. None of the included cases had intravitreal dexamethasone implant. This yielded a relatively young age group in the current study. Males were slightly higher than females. Steroid-induced glaucoma has no gender or racial predilection [21]. The reported incidence of SIG may be underestimated because many cases went undiagnosed especially patients receiving systemic steroids. Most of these patients do not follow up their intraocular pressure. Also, frequent use of topical steroid eye drops without advice from the doctor to relieve chronic eye symptoms leads to increased incidence of SIG among our patients in Egypt. The reported incidence of marked increase of IOP after 4 to 6 weeks of using topical corticosteroids is 5–6% [22].

Nonpenetrating glaucoma surgery avoids the complications of trabeculectomy and yields comparable effect on lowering IOP especially if combined with other step, for example, use of mitomycin C, trabeculotomy, collagen implant, viscocanalostomy [23], or Ex-PRESS mini shunt [24] (Alcon Inc., Fort Worth, TX). Trabeculectomy has many reported complications such as choroidal effusion, hypotony maculopathy, hyphema, blebitis, and bleb leakage. In 2016, a published study reported an incidence of 30% of hypotony in the follow-up period of trabeculectomy [25].

The current study focuses on reporting 4-year follow-up results of primary deep sclerectomy in SIG patients. The limitations of the study were the retrospective nature and lack of a control group. It had the advantages of long-term follow-up and focusing on steroid-induced glaucoma patients in a relatively young age group. All the NPDS surgeries were performed by the same surgeon with a reproducible technique to minimize variations among different patients. The depth of dissection and the anterior extension of dissection can vary the postoperative results.

In all included eyes, mitomycin C and a collagen implant were used during NPDS to enhance the IOP lowering effect and to prevent fibrosis that would obstruct the aqueous outflow. The relatively younger age population has higher healing capability with more tendencies to subconjunctival fibrosis and expected more difficult control of IOP without augmentation of the effect of NPDS. Also, the high preoperative mean IOP necessitated higher percentage of reduction to reach optimum target IOP levels. In the current study, we achieved 60–64% reduction from the high preoperative IOP levels that were maintained during the 4 years of follow-up periods. Some authors did not use mitomycin C with NPDS like Shaarawy et al. [26] while others (similar to the current study) used mitomycin C 0.2 mg/ml for two minutes [27].

As regards the safety of the procedure among the included patients, no major complications were detected and not a single case of hypotony. Shaarawy et al. [26] reported that no patient developed a shallow/flat anterior chamber, endophthalmitis, or surgery-induced cataract in a 5-year follow-up report of NPDS with collagen implant. During record scanning, 5 cases were transformed into full penetrating trabeculectomy due to accidental perforation during dissection and those cases were excluded from the study. During the follow-up period, the mean CDVA did not statistically differ from the preoperative levels despite a minor drop noticed at 3rd and 4th years. Five patients lost 1 line of CDVA, and none of the patients lost ≥2 lines. This may be attributed to the development of a complicated cataract due to steroids itself or progression of the glaucoma. However, visual acuity is not a good indicator for the success or the follow-up of a glaucoma surgery because there might be other confounding factors affecting the vision. Harju et al. [28] reported long-term results of NPDS with or without mitomycin C in normal tension glaucoma. They also did not report any major complications. They reported that 4 out of 37 eyes had lost 2 or more lines of CDVA, and they reported that those eyes had other pathologies that explained the drop in vision.

At the end of the follow-up period of 4 years, complete success rate was 56.7% (34 eyes). This represented eyes with IOP ≤ 21 mmHg with no additional medications or interventions. Qualified success rate was 70% (42 eyes). This represented eyes with IOP ≤ 21 mmHg with topical medications with no additional interventions. Unlike others [29–31], we considered further interventions such as needling a surgical failure and excluded those eyes from the complete success. They considered needling as an enhancement to reestablish a good filtration in a preexisting bleb. However, there are no standards for this subject as Heuer et al. [32] opposed this like our study. When the postoperative IOP level was unsatisfactory (either >21 mmHg or there was a progression in the mean deviation of the visual field defect), an intervention in the form of YAG laser goniopuncture, needling, or ab interno revision was resorted to. The authors preferred ab interno revision over needling because the latter is a blind procedure, whereas in ab interno revision, the surgeon can see what he/she is doing. When needling failed once, we resorted to ab interno revision. Koukkoulli et al. [33] reported 64% 1 year success after needling post NPDS and 40% 5-year success. The rate of YAG laser goniopuncture was minimal in the current study (5 eyes representing 8.3%) which was much lower than that reported in other studies [26]. Anand and Pilling [34] reported an annual rate of YAG laser goniopuncture around 20%. Topical medications were tried first to control IOP before the decision to intervene.

The authors preferred ab interno revision under vision and subconjunctival 5 fluorouracil injections over YAG laser goniopuncture because in many cases the trabeculo-Descemet's membrane could not be easily visualized besides the unfamiliarity of this technique to us. Also, YAG laser goniopuncture might lead to iris incarceration into the trabeculo-Descemet's membrane as a complication in up to 32% of the cases [28, 34].

Eksioglu et al. [35] evaluated the outcomes of Ahmed glaucoma valve (AGV) in the management of elevated IOP secondary to steroid use. They retrospectively evaluated 9 eyes of 5 patients. The mean age of their included patients was comparable to the current study (25.0 ± 8.3 years), and the mean follow-up period was 38.4 ± 13.2 months. The preoperative mean IOP level was 41.0 ± 8.3 mmHg and 12.8 ± 4.2 mmHg at 48th month. The degree of IOP reduction using AGV was comparable to our results using deep sclerectomy. They reported a decrease of mean topical antiglaucomatous medications used from 2.8 ± 0.4 preoperative to 0.4 ± 0.9 at 48 months postoperative, which was also comparable to our results. Complete success rate was obtained in 7 (77%) eyes with no cases with failure. They reported 2 eyes with hypotony with choroidal detachment in one eye. Hyphema was reported in one eye. In our study, we had no case of hypotony despite the larger number of included patients in the current study. Ahmed glaucoma valve can provide comparable success as regards IOP management but somehow more liable to complication especially hypotony and the related complications like choroidal detachment. Nonpenetrating deep sclerectomy appears to be a more safe option with similar effect.

Trabeculectomy is considered the gold standard surgical procedure for most glaucoma cases. However, it is associated with a high incidence of early and late complications and surgical failure often observed over time [36]. Honjo et al. [37] investigated the effect of trabeculectomy on the eyes suffering from SIG. They retrospectively analyzed the results of 14 eyes of 7 patients with an average follow-up period of 60.6 ± 33.5 months. They concluded that intraocular pressure was well controlled ≤ to 21 mmHg at the final examinations. However, they reported the usual hypotony-related and bleb-related complications associated with trabeculectomy.

In conclusion, primary nonpenetrating deep sclerectomy is a safe and an effective method of treating eyes with steroid-induced glaucoma. No major complications were encountered. After 4 years of follow-up, complete success rate was 56.7%, and qualified success rate was 70%.

## Figures and Tables

**Table 1:** The mean preoperative and postoperative intraocular pressure values among the study group.

	Intraocular pressure (mmHg) (mean ± SD)	*P* value
Preoperative	34.2 ± 6.9	N/A
At 6 months	12.3 ± 2.1	0.001^∗^
At 12 months	12.3 ± 2.6	0.001^∗^
At 24 months	12.8 ± 3.2	0.001^∗^
At 36 months	13.3 ± 3.1	0.001^∗^
At 48 months	13.6 ± 2.8	0.001^∗^

^∗^Significant using paired *t*-test to compare with mean preoperative levels.

**Table 2:** The mean preoperative and postoperative values for topical antiglaucoma medications required among the study group.

	Medications required (mean ± SD)	*P* value
Preoperative	2.47 ± 0.51	N/A
At 6 months	0.07 ± 0.25	0.001^∗^
At 12 months	0.13 ± 0.34	0.001^∗^
At 24 months	0.20 ± 0.40	0.001^∗^
At 36 months	0.27 ± 0.45	0.001^∗^
At 48 months	0.33 ± 0.48	0.001^∗^

^∗^Significant using paired *t*-test to compare with mean preoperative levels.

**Table 3:** The mean best-corrected visual acuity and the visual field defect mean deviation among the study group.

	CDVA (mean ± SD)	*P* value	Visual field defect mean deviation (dB) (mean ± SD)	*P* value
Preoperative	0.37 ± 0.13	N/A	−12.51 ± 7.33	N/A
At 6 months	0.39 ± 0.11	0.112	−12.54 ± 7.12	0.210
At 12 months	0.35 ± 0.14	0.121	−12.65 ± 7.45	0.122
At 24 months	0.39 ± 0.12	0.111	−13.51 ± 8.76	0.038^∗^
At 36 months	0.42 ± 0.12	0.089	−14.77 ± 7.13	0.001^∗^
At 48 months	0.44 ± 0.15	0.067	−15.51 ± 6.83	0.001^∗^

^∗^Significant using paired *t*-test to compare with mean preoperative levels. CDVA: corrected distance visual acuity; dB: decibel.

## Abbreviations

SIG: Steroid-induced glaucoma
IOP: Intraocular pressure
TM: Trabecular meshwork
NPDS: Nonpenetrating deep sclerectomy
SC: Schlemm's canal
YAG: Yttrium aluminum garnet
CDVA: Corrected distance visual acuity
dB: Decibel
AGV: Ahmed glaucoma valve.
